# Pretraining effective T5 generative models for clinical and biomedical applications

**DOI:** 10.1371/journal.pone.0342610

**Published:** 2026-04-17

**Authors:** Saad Althabiti, Chuming Chen, Sultan Alrowili, Cathy Wu, K. Vijay-Shanker

**Affiliations:** 1 Department of Health Informatics, King Saud bin Abdulaziz University for Health Sciences, Riyadh, Saudi Arabia; 2 King Abdullah International Medical Research Center, Riyadh, Saudi Arabia; 3 Ministry of the National Guard - Health Affairs, Riyadh, Saudi Arabia; 4 Center for Bioinformatics and Computational Biology, University of Delaware, Newark, Delaware, United States of America; 5 Department of Computer & Information Sciences, University of Delaware, Newark, Delaware, United States of America; 6 IBM Research, Riyadh, Saudi Arabia; PLOS: Public Library of Science, UNITED KINGDOM OF GREAT BRITAIN AND NORTHERN IRELAND

## Abstract

This paper presents a study of the impact of corpus selection and vocabulary design on the performance of T5-based language models in clinical and biomedical domains. We introduce five different T5-EHR models, each pretrained from scratch using different combinations of clinical and biomedical corpora alongside domain-specific vocabularies. We evaluated these models across a variety of clinical and biomedical tasks to quantify the impact of pretraining data and vocabulary tokenization choices on downstream performance. Our findings reveal the importance of aligning both pretraining corpus and vocabulary with the target domain. Models pretrained exclusively on clinical data achieve superior performance on clinical tasks, while adding biomedical data contributes only marginal gains in most cases, with a few exceptions. Similarly, the choice of vocabulary significantly influences model performance, with clinical-specific vocabularies outperforming general biomedical vocabularies in tasks requiring a deeper understanding of clinical language. Also, the T5 generative models perform competitively with state-of-the-art discriminative models on several biomedical benchmarks, demonstrating strong generalization to biomedical domain. Overall, these results emphasize that task-specific selection of corpus and vocabulary is essential for optimizing model performance in clinical and biomedical natural language processing (NLP).

## 1. Introduction

Natural Language Processing (NLP) has become an essential tool in different areas enabling machines to understand and even generate human language. While general-purpose NLP models have achieved remarkable success across several tasks, there is a growing need for domain-specific models that can handle the unique linguistic features and complexities of specialized fields, such as clinical and biomedical domains. Traditionally, discriminative models, which focus on predicting labels for given inputs, have dominated tasks like classification, named entity recognition, and relation extraction. However, recent advances in generative models have shown that these models can achieve performance that are comparable to, or even better than, discriminative models on many tasks [1–3]. In addition, generative models, particularly those based on the transformer architecture, have demonstrated their ability to not only perform traditional NLP tasks but also to handle more complex scenarios, such as summarization, question answering, and text generation.

Moreover, the choice of corpus and vocabulary plays a crucial role in impacting the performance of NLP models, especially in domain-specific applications like clinical and biomedical text processing [4]. The corpus used for pretraining impacts the model's understanding of domain-specific language, as it determines the scope of the model's exposure to relevant terminology, syntax, and contextual usage. Similarly, vocabulary selection significantly affects how well a model can process and generate domain-specific terms. By aligning both corpus and vocabulary to the targeted domain, models can achieve improved accuracy and robustness, particularly in tasks that require deep understanding of domain-specific context, thus enhancing their performance in clinical and biomedical tasks.

Our study explores and adapts generative models to specialized domains, motivated by recent findings showing that these models can achieve performance comparable to, or even better than, discriminative models across various NLP tasks. Additionally, unlike discriminative models, generative models such as T5 function in a sequence-to-sequence (seq2seq) framework, which allows them to both understand input text and generate new outputs, making them particularly valuable for clinical applications that require text generation tasks such as summarization and report writing. Despite their potential, relatively few generative models have been specifically adapted to the clinical and biomedical domains, where the language differs significantly from general domain text in terms of vocabulary, structure, and style. This gap highlights the need for more domain-adapted generative models that can better capture the unique characteristics of clinical and biomedical language.

In this work, we present a study and analysis of the impact of corpus selection and vocabulary choice on the performance of T5-based models in clinical and biomedical domains. We developed five variants of T5-EHR models, each trained on distinct combinations of clinical and biomedical corpora, using vocabularies derived from either domain. Our primary focus is to analyze how the integration of diverse data sources and optimization of specific vocabulary selections influence the models’ ability to perform clinical tasks, while also assessing their generalization to biomedical tasks. We selected the T5 model as the base architecture for our models for two key reasons: (1) recent studies have shown that these models can achieve results that are comparable to, or even better than, discriminative models on many tasks [1–3], and (2) T5, as a sequence-to-sequence (seq2seq) model, includes an additional decoder layer that enables it to generate new text. This capability allows T5 to manage generative clinical tasks, like clinical summarization, that discriminative models are not built to handle. Furthermore, our work also differs from recent large language models (LLMs) in its practical design. Many existing generative models are large and resource-intensive, making them costly to train and deploy. In contrast, we adopt the T5-Base architecture as a computationally efficient alternative that is better suited for clinical settings with limited computational resources.

Although recent advances have led to strong generative models for biomedical NLP, most of these efforts have concentrated on structured biomedical literature rather than on real-world clinical text. Generative models pretrained directly on electronic health records remain relatively limited, despite the distinct challenges posed by clinical narratives, including abbreviations, fragmented structure, and context-dependent language. This distinction motivates our focus on systematically studying corpus and vocabulary choices for generative models in clinical settings, while also examining how such models generalize to biomedical tasks.

To guide our study, we investigate a series of targeted questions that examine the impact of corpus and vocabulary choices on model performance. Specifically, we explore whether biomedical literature alone is sufficient to support clinical tasks, and whether real clinical data by itself can provide a strong foundation for clinical NLP. Also, we assess the influence of vocabulary alignment by comparing models using clinical versus biomedical vocabularies when trained on mixed corpora. Additionally, we evaluate whether expanding the training data with a large corpus improves performance. Finally, we examine whether domain-adapted generative models like T5-EHR can perform competitively with discriminative models on biomedical benchmarks.

To summarize the contributions of this work, we highlight the following key contributions:

1. We present five novel variants of the T5-EHR model, all pretrained from scratch using distinct combinations of clinical and biomedical corpora (MIMIC, PubMed, and PMC) and domain-specific vocabularies, enabling a systematic investigation of corpus and vocabulary design choices.
2. We provide a comprehensive empirical evaluation of these variants across multiple clinical and biomedical NLP tasks, including inference, relation extraction, question answering, and biomedical relation benchmarks, to assess how corpus and vocabulary design affect downstream performance.
3. We demonstrate that domain-aligned pretraining, where both the corpus and vocabulary are matched to the target domain task, yields substantially better performance on clinical tasks compared to generic or mismatched configurations.
4. We show that our T5‑EHR v5, not only outperforms prior generative models, but also achieves performance comparable to, and in some cases exceeding, strong discriminative baselines, thereby confirming that generative models can be competitive in clinical and biomedical settings.
5. We plan to release the full set of pretrained models, model weights, tokenizers, and configuration files under controlled access (via PhysioNet) for the research community, enabling reproducibility and further development.
6. Finally, we provide practical guidance on selecting appropriate pretraining corpus and vocabulary configurations depending on the target task, offering a roadmap for building domain-specific generative models for healthcare NLP.

## 2. Related work

Literature on domain-specific language models has grown significantly in recent years, particularly in the biomedical and clinical domains. Pretrained language models such as BERT [5], GPT-3 [6], and T5 [2] have demonstrated remarkable performance across a range of NLP tasks. However, their effectiveness lessens when applied to domain-specific texts like clinical notes or biomedical literature due to the unique vocabulary and writing styles present in these domains [7].

### 2.1. Discriminative and generative language models

In addition to generative models, there has been significant progress in developing discriminative models for clinical and biomedical applications. Discriminative models are in general designed for tasks such as classification, named entity recognition, and relation extraction, where the goal is to predict labels or outcomes based on input features. These models have shown strong performance across various tasks, contributing valuable insights to the field.

Generative models have significantly shaped the NLP field by enabling the generation of coherent and contextually relevant text. A significant contribution in this field is the Text-to-Text Transfer Transformer (T5), introduced by [2]. T5 redefines NLP tasks as text-to-text problems, leveraging an encoder-decoder architecture. This approach demonstrated high performance across a range of benchmarks, setting the stage for generative model advancements. Similarly, BART (Bidirectional and Auto-Regressive Transformer) [8] uses an encoder-decoder architecture. BART proved especially powerful for text generation tasks (e.g., abstractive summarization) while still performing competitively with encoders like RoBERTa on understanding tasks.

Moreover, Generative Pre-trained Transformer (GPT) models are decoder-only LMs trained to predict the next word in large-scale text. GPT-4o [9], released by OpenAI, extends generative capabilities to multimodal inputs, including text and images, showcasing the trend toward integrating diverse data types. Additionally GPT‑4.5 [10], which was built on GPT‑4o, scales pre-training further and it is the largest and most knowledgeable model released by OpenAI.

### 2.2. Adaptation to clinical and biomedical domain

ClinicalBERT [11] and BioBERT [12] were early domain-specific BERT adaptations, pretrained on MIMIC-III, PubMed, and PMC to enhance task performance in clinical and biomedical settings. Extensions like Bio-ClinicalBERT and Bio-DischargeSummaryBERT combine biomedical and clinical corpora to boost accuracy on classification and NER tasks. Furthermore, PubMedBERT [7] demonstrated that pretraining biomedical language models from scratch, using only in-domain PubMed abstracts and vocabulary, significantly improved performance across multiple biomedical NLP tasks included in the BLURB benchmark, emphasizing the importance of in-domain pretraining and vocabulary design for biomedical applications.

Moreover, [4] investigates the performance of various pretrained language models on biomedical and clinical NLP tasks. Also, they introduced new model variants of RoBERTa adapted specifically for biomedical and clinical text. The study highlights the effectiveness of specialized models and vocabularies in enhancing downstream performance in biomedical NLP tasks.

The development of generative models specifically tailored to the clinical domain has been relatively limited, while most research efforts focus on general or biomedical language models. Nevertheless, there have been some notable efforts in developing generative models for the clinical domain, for example, ClinicalT5 [13] addresses this gap by further pretraining SciFive models on MIMIC-III notes, showing improved performance over general T5 across clinical tasks. Similarly, [3] pretrained T5-Base and T5-Large from scratch on MIMIC-III and MIMIC-IV notes, demonstrating that even smaller clinical models can outperform large general-purpose models when trained on in-domain data. These results substantiate the need for developing and using specialized clinical models to address the unique challenges of clinical text processing, especially in resource-constrained healthcare settings.

Recent studies have also examined the efficiency and domain trade-offs of T5-based models in clinical and biomedical NLP. For example, [14] systematically evaluate clinical T5 variants and show that domain-specialized T5 models offer modest but consistent gains under certain conditions, while remaining computationally lightweight. Similarly, [15] show that efficiency-oriented design choices, such as instance selection and multi-task learning built on SciFive, can significantly reduce training cost while maintaining strong performance on biomedical benchmarks. Other work [16] highlights that domain adaptation does not uniformly improve performance across tasks, and that careful corpus and vocabulary alignment is often more impactful than scale alone. These studies reinforce our design choice to adopt T5-Base as a practical and reproducible backbone, enabling domain adaptation under realistic computational constraints rather than relying on extremely large, resource-intensive biomedical LLMs.

Beyond language-based models, recent work has also demonstrated the effectiveness of deep learning in other healthcare modalities, such as medical imaging. For example, [17] proposed a convolutional neural network with attention mechanisms for multiple sclerosis lesion classification using MRI data, achieving strong diagnostic performance. While this work operates in a fundamentally different setting, medical image analysis rather than natural language processing, it highlights the broader impact of domain-specific deep learning models in healthcare and provides complementary context to our focus on generative language models for clinical and biomedical text.

In the biomedical domain, [1] introduced SciFive, a biomedical adaptation of T5, pretrained on PubMed and PMC articles, achieving strong results across multiple biomedical tasks. Furthermore, BioBART [18] extends BART for the biomedical domain, showing consistent improvements on tasks like summarization, entity linking, and QA.

While prior generative language models advanced biomedical or clinical pretraining, our work introduces several key innovations that distinguish it from all earlier efforts.

First, SciFive [1] was not pretrained from scratch: it was initialized from the general‑domain T5‑Base checkpoint and was then further trained on combinations of C4, PubMed abstracts, and PMC articles. This design embeds a strong general-language prior and optimizes primarily for biomedical/general-literature text. By contrast, our models were pretrained from scratch and explored targeted corpus and vocabulary alignment (clinical vs. biomedical), rather than inheriting general-domain information.

Second, ClinicalT5 [13] was pretrained solely on MIMIC-III clinical notes only, but importantly it was initialized from the SciFive-PubMed-PMC checkpoint. This means its learned representations are influenced heavily by biomedical literature and even C4 text. Our work instead isolates this effect: by pretraining from scratch on cleanly defined corpus and vocabulary configurations.

Third, Clinical‑T5 [3] attempted a scratch-trained model; however, this model was trained for fewer pretraining steps and used a lower-cased vocabulary. In contrast, our models are trained fully (500K steps) on TPUv3-128 without such interruptions, and with correct cased vocabularies aligned to each domain.

In summary, our contribution is not simply another T5-based clinical model, but a systematic, controlled study of how corpus composition and vocabulary design interact to influence downstream performance. We also compare our T5-EHR models with these models on clinical and biomedical benchmarks in the Results section. We provide five fully pretrained variants, enabling the community to choose and build on the configuration that best suits their use case.

## 3. Methods: Pretraining of our models

### 3.1. Corpora and vocabulary

We pretrain our models using clinical and biomedical corpora, specifically MIMIC-III, MIMIC-IV, PubMed Abstracts, and PMC Full-Text Articles. These corpora provide a broad representation of clinical narratives and biomedical literature. MIMIC-III and MIMIC-IV consist of real clinical notes written by healthcare providers during patient care, characterized by informal language, abbreviations, and fragmented writing. In contrast, PubMed and PMC are biomedical collections that include both research-focused articles and medical texts discussing treatments, diseases, and clinical practices, written in a more formal and structured style. This difference in writing style is important because clinical notes reflect real-world patient care, while biomedical texts are more descriptive and research oriented.

Additionally, we constructed two SentencePiece-based vocabularies: one from MIMIC and one from PubMed data. Each vocabulary contains 30,000 tokens. More details about the corpus and preprocessing steps are provided in section A in S1 Appendix. Our vocabulary pipeline is unique in that we use the SentencePiece algorithm trained directly on each model’s pretraining corpus (e.g., MIMIC, PubMed). This ensures full alignment between the tokenizer and the domain language. Unlike prior work that reused general-domain vocabularies, our approach is entirely corpus-specific and reproducible, others can easily replicate or adapt it to new domains. All tokenizer files are included in our release to support future reuse.

### 3.2. Architecture

To assess and meet the demands of the clinical domain, we pretrained five distinct T5 models from scratch using a variety of corpora derived from clinical and biomedical texts. Table 1 details each of these models. All five models are based on the T5 base architecture, consisting of approximately 110 million parameters in the encoder and around 220 million parameters in total (encoder and decoder combined), with 768 hidden units, 12 encoder layers, and 12 decoder layers. This architecture employs a fully connected transformer model that features both an encoder and a decoder, making it particularly effective for generative tasks. During pretraining, T5 uses a span corruption objective (also called masked span prediction), where random spans of text are replaced with special tokens and the model is trained to reconstruct the missing spans. This differs from BERT’s pretraining tasks, which include masked language modeling (MLM) and next sentence prediction (NSP). The encoder processes the corrupted input sequence and converts it into contextual representations, while the decoder uses these representations to predict the masked spans.

**Table 1:** T5-EHR models.

Models	Corpora	#Tokens	Vocab	Vocab Size
**T5-EHR – v1**	PubMed	~ 4.5B	PubMed	30K
**T5-EHR – v2**	MIMIC	~ 2B	MIMIC	30K
**T5-EHR – v3**	MIMIC + PubMed	~ 6.5B	PubMed	30K
**T5-EHR – v4**	MIMIC + PubMed	~ 6.5B	MIMIC	30K
**T5-EHR – v5**	MIMIC + PubMed + PMC	~ 20B	MIMIC	30K

In addition to methodological contributions, our work is motivated by practical considerations that distinguish it from recent trends in large language model development. Many existing LLMs rely on billions of parameters and require substantial computational resources for training and deployment, making them costly and often impractical for real-world clinical environments. In contrast, our choice of T5-Base architecture reflects a computationally efficient and realistic alternative. This design enables training and fine-tuning using more accessible infrastructure and better aligns with the resource constraints commonly faced by clinical institutions. As a result, our approach prioritizes both domain adaptation and feasibility, highlighting a practical pathway for deploying generative models in clinical NLP settings.

**Fig 1:**
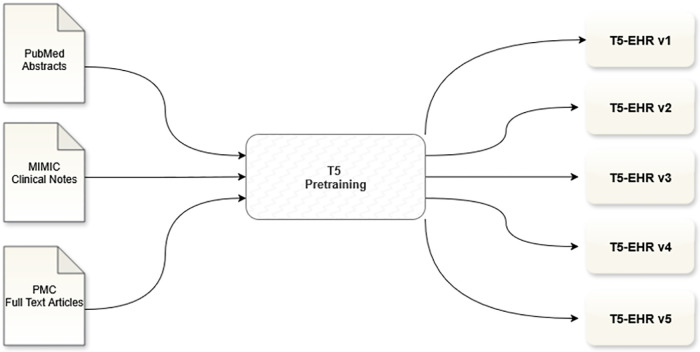
Pretraining pipeline for T5-EHR models. As shown, five distinct versions of **T5-EHR** (v1–v5) were developed, each reflecting different choices in corpus selection and vocabulary design. This design allowed us to systematically study the effect of clinical versus biomedical corpora and the impact of vocabulary alignment on downstream performance.

Because T5 is trained under a text-to-text framework, downstream tasks, including classification tasks, are formulated as generation problems where the model is prompted to output a label or structured text given an input context. This design allows the same model to flexibly handle classification, translation, summarization, question answering, and other tasks within a unified architecture. Table 1 and Fig 1 provide an overview of the pretrained models.

### 3.3. Pretraining setup

All our five T5 models are pre-trained for 500K steps with a batch size of 128 tokens. We leveraged TPUv3-128, a high-performance distributed computing environment optimized for large-scale deep learning workloads, to efficiently train each model. Our pretraining pipeline was implemented using the t5x library [19], which provides a flexible and scalable framework for training T5 models with several optimizations processes. Because of the availability of high-performance TPUs and the use of optimized training strategies, the pretraining process was completed significantly faster than initially estimated, with each model trained in approximately three days.

## 4. Evaluation setup

We selected a diverse set of downstream tasks within the clinical and biomedical domains to thoroughly evaluate the performance of our models. By focusing on both clinical and biomedical tasks, we aimed to demonstrate the adaptability and effectiveness of our models in handling various types of data and domain specific tasks.

### 4.1. Evaluation tasks

To evaluate the effectiveness of our models in both clinical and biomedical settings, we conduct experiments on a diverse set of benchmark tasks. The first four tasks (natural language inference, relation extraction, question answering, and classification) are taken to be clinical tasks as they are based on datasets derived from Electronic Health Records (EHRs), ensuring that the evaluation closely reflects real-world clinical text. In contrast, the remaining three tasks are biomedical tasks derived from PubMed and are well-established, widely used in prior biomedical NLP research, and cover diverse types of biomedical knowledge. We use them to evaluate whether domain-adapted generative models like T5-EHR can generalize to biomedical tasks after being trained primarily for clinical NLP. Table 2 shows a summary of the datasets used. More detailed descriptions can be found in section B in S1 Appendix.

**Table 2:** Summary of our selected tasks.

Task Name	Domain	Task	Train Records	Dev Records	Test Records
MedNLI	Clinical	Natural Language Inference	11,232	1395	1422
i2b2-2010 RE	Clinical	Relation Extraction	22,160	874	43,000
RadQA	Clinical	Question-Answering	4878	656	614
CLIP	Clinical	Multi-Labeling	77,015	15,294	15,643
ChemProt	Biomedical	Relation Extraction	18,035	11,268	15,745
DDI-2013	Biomedical	Relation Extraction	25,296	2496	5716
GAD	Biomedical	Relation Extraction	4261	535	534

### 4.2. Hyperparameter tuning

We performed hyperparameter tuning using the T5-EHR v1 model and applied the optimal settings consistently across all models to ensure fair comparisons. We summarize the selected hyperparameter configurations in Table 3, with additional details provided in section C in S1 Appendix. We experimented with different learning rates, batch sizes, epochs, and sequence lengths. A learning rate of 1e-4 and smaller batch sizes consistently led to better performance. Most tasks converged within 10–20 epochs, while RadQA required 60 epochs. We selected the best sequence length per task and used development set performance to guide all hyperparameter tuning.

**Table 3:** Summary of the hyperparameter tuning.

Task Name	Learning Rate	Batch Size	Epoch	Input Length	Run-Time
MedNLI	1e-4	8	10	256	~ 00:22:45
i2b2-2010 RE	1e-4	8	10	256	~ 00:55:46
RadQA	1e-4	8	60	1024	~ 02:12:37
CLIP	1e-4	8	10	256	~ 02:25:24
ChemProt	1e-4	8	10	512	~ 00:51:16
DDI-2013	1e-4	8	20	128	~ 01:26:14
GAD	1e-4	8	20	128	~ 00:16:04

## 5. Results and discussion

In this section, we present the experimental results and analyze how different corpus and vocabulary configurations influence the performance of T5-EHR models across clinical and biomedical tasks. Our goal is to understand the role of domain alignment in pretraining and how it affects generalization to both clinical notes and biomedical text.

### 5.1. Impact of domain selection

We begin our experiments by comparing T5-EHR v1 and T5-EHR v2, which allows us to directly evaluate how clinical corpus selection and vocabulary alignment influence model performance on clinical tasks (Table 4).

**Table 4:** T5-EHR v1 vs T5-EHR v2.

Models	Corpora	Vocab	MedNLI [Accuracy]	i2b2 RE [F1-Score]	RadQA [F1-score] | [EM]		CLIP [Micro F1] | [Macro F1]	
T5-EHR – v1	PM	PM	80.2	70.6	63.0	42.7	76.1	63.3
T5-EHR – v2	M	M	**86.4**	**76.9**	**72.1**	**53.3**	**77.4**	**65.5**

PM: PubMed, M: MIMIC.

Across all clinical tasks, T5-EHR v2 shows clear and consistent improvements over T5-EHR v1, highlighting the effectiveness of using clinical data and vocabulary for domain-specific model pretraining. The model’s strong performance supports our hypothesis that alignment with the language and structure of electronic health records is critical for clinical NLP. Given its strong and consistent performance, we select T5-EHR v2 as the baseline model for subsequent comparisons and further analysis.

### 5.2. Impact of adding biomedical corpus and vocabulary

Having shown the benefit of pretraining on MIMIC alone for clinical tasks, we now examine whether incorporating clinical data into the PubMed abstracts corpus improves performance. We also explore how vocabulary selection interacts with corpus configuration in this setting. This allows us to explore whether performance gains can be achieved through corpus combination, and how vocabulary choice influences the outcome.

As shown in Table 5, T5-EHR v4 slightly outperforms both T5-EHR v2 and T5-EHR v3 across most tasks, indicating that combining MIMIC and PubMed can be beneficial, but only when the vocabulary remains aligned with clinical language. T5-EHR v3, which uses a PubMed-derived vocabulary, shows mixed performance despite having access to the same corpora. These results suggest that the best performance is achieved on the larger corpora obtained by combining clinical and biomedical corpora with a clinical vocabulary. Based on these results, we select T5-EHR v4 for further analysis.

**Table 5:** T5-EHR v2 vs T5-EHR v3 vs T5-EHR v4.

Models	Corpora	Vocab	MedNLI [Accuracy]	i2b2 RE [F1-Score]	RadQA [F1-score] | [EM]		CLIP [Micro F1] | [Macro F1]	
T5-EHR – v2	M	M	86.4	76.9	72.1	53.3	77.4	65.5
T5-EHR – v3	M + PM	PM	85.7	76.7	**74.5**	**56.0**	77.2	66.0
T5-EHR – v4	M + PM	M	**86.5**	**77.2**	72.3	51.2	**77.6**	**68.0**

PM: PubMed, M: MIMIC.

### 5.3. Impact of adding PMC corpus

Having shown that the PubMed inclusion improves the models when the vocabulary remains aligned with clinical language, we now evaluate whether adding a larger and more diverse biomedical corpus like PMC improves model performance. This analysis focuses on whether the inclusion of PMC contributes meaningful gains beyond the combination of MIMIC and PubMed, or whether addition of a large, diverse dataset doesn’t contribute to clinical domain tasks.

As shown in Table 6, T5-EHR v5, which incorporates PMC in addition to MIMIC and PubMed, does not show meaningful improvements over T5-EHR v4 and slightly underperforms on most tasks’ metrics. This suggests that although PMC adds volume and diversity, its content, typically composed of structured and formal academic biomedical writing, may not align well with the style and terminology found in real-world clinical notes. Therefore, for clinical NLP applications, the precision and relevance of training data are crucial, and expanding the corpus with less directly relevant data can introduce noises that degrade the model performance. Based on the results, we retain T5-EHR v4 as the best-performing model for clinical tasks.

**Table 6:** T5-EHR v4 vs T5-EHR v5.

Models	Corpora	Vocab	MedNLI [Accuracy]	i2b2 RE [F1-Score]	RadQA [F1-score] | [EM]		CLIP [Micro F1] | [Macro F1]	
T5-EHR – v4	M + PM	M	**86.5**	**77.2**	**72.3**	**51.2**	**77.6**	**68.0**
T5-EHR – v5	M + PM + P	M	84.4	76.9	71.5	51.1	**77.6**	66.2

PM: PubMed, M: MIMIC, P: PMC.

Finally, Table 7 compares the performance of our best model, T5-EHR v4, against other generative models with the same settings. Additionally, we included results from two widely used discriminative models in the field for reference.

**Table 7:** T5-EHR-v4 models vs other Models.

Models	Corpora	Vocab	MedNLI [Accuracy]	i2b2 RE [F1-Score]	RadQA [F1-score] | [EM]		CLIP [Micro F1] | [Macro F1]	
T5-EHR – v4	M + PM	M	86.5	77.2	72.3	51.2	77.6	68.0
Clinical-T5 ^1^	M	M	85.1	75.4	70.5	52.5	72.8	51.1
ClinicalT5 ^2^	M + PM + P*	PM	83.4	74.8	72.4	47.8	76.3	61.4
SciFive^3^	C4 + PM**	G	83.4	73.5	69.5	50.4	75.9	61.1
BioRoBERTa^4^	M3 + PM + P	PM	87.1	75.0	--	--	--	--
ClinicalBERT^5^	M3***	G	81.8	74.0	62.6	45.7	77.6	64.9

PM: PubMed M: MIMIC P: PMC G: General NR: Not Reported.

1 [3].

2 [13].

3 [1].

4 (Lewis et al., 2020) [We rename the model (RoBERTa-base-PM-M3-Voc) to be BioRoBERTa.].

5 [11].

* Initialized the weights from the SciFive-PubMed-PMC model.

** Initialized the weights from T5-Base model.

*** Initialized the weights from BioBERT model.

BioRoBERTa Large results for RadQA: F1 = 75.1 & EM = 60.3 | CLIP: Micro = 79.2 & 67.7

Compared to other generative models, T5-EHR v4 achieves the strongest overall performance across all clinical tasks. It shows the highest scores on MedNLI, i2b2 RE, and CLIP, and remains competitive on RadQA. These results highlight the effectiveness of domain-specific pretraining on MIMIC and the inclusion of PubMed using clinically aligned vocabulary. Although T5-EHR v3 is not shown in this table, it achieved the best RadQA performance among all generative models, as detailed earlier in Table 3, further demonstrating the importance of evaluating both corpus and vocabulary combinations.

Further, we also compare T5-HER-v4 with BioRoBERTa and ClinicalBERT, both strong and widely used discriminative models as baselines. These models have been widely adopted in clinical NLP due to their robust performance on various benchmarks. It is important to note that the reported results for BioRoBERTa on RadQA and CLIP come from the *large* variant, making them not directly comparable to the base-sized T5-EHR models. Nevertheless, the inclusion provides useful context, and the generative T5-EHR v4 demonstrates competitive performance, highlighting its ability across different clinical tasks.

### 5.4. Biomedical tasks

Having evaluated the models on clinical tasks, we now focus on evaluating their performance on biomedical tasks. This analysis aims to assess how well our models, which are trained on various corpora and vocabularies, generalize to not only broader biomedical applications but also to text coming from research articles.

As shown in Table 8, performance improves progressively as the training corpus incorporates more biomedical data. T5-EHR v5, which was trained on MIMIC, PubMed and PMC with MIMIC vocab, achieves the highest scores on ChemProt and DDI and performs competitively on GAD. We note that the GAD test set is considerably smaller than the other two datasets, and its results should be interpreted with some caution. Based on its overall performance, we select T5-EHR v5 as the model for comparison with other existing generative and discriminative baselines.

**Table 8:** Biomedical tasks.

Models	Corpora	Vocab	ChemProt [F1-Score]	DDI [F1-Score]	GAD [F1-Score]
T5-EHR – v1	PM	PM	76.1	76.4	75.5
T5-EHR – v2	M	M	77.4	79.4	76.2
T5-EHR – v3	M + PM	PM	77.2	81.4	**82.6**
T5-EHR – v4	M + PM	M	77.6	81.5	78.7
T5-EHR – v5	M + PM + P	M	**77.6**	**83.7**	81.5

PM: PubMed M: MIMIC P: PMC.

Next, we compare the performance of T5-EHR v5 with several existing generative and widely used discriminative models. These models vary in architecture, training corpora, and vocabulary types, providing a comprehensive view of performance across biomedical tasks (Table 9).

**Table 9:** Biomedical tasks.

Models	Corpora	Vocab	ChemProt [F1-Score]	DDI [F1-Score]	GAD [F1-Score]
T5-EHR – v5	M + PM + P	M	77.6	83.7	81.5
Clinical-T5 [3]	M	M	52.3	64.3	74.0
ClinicalT5 [13]	M + PM + P**	PM	76.6	78.7	81.1
SciFive [1]	C4 + PM***	G	76.8	82.0	81.0
BioRoBERTa [4]	M3 + PM	PM	NR	81.0	82.2
BioLinkBERT [20]	PM	PM	77.6	82.7	84.4
BioBERT [12]	PM + P*	G	75.1	NR	81.5
BioMedBERT [7]	PM	PM	77.2	82.4	84.0

PM: PubMed M: MIMIC P: PMC G: General NR: Not Reported.

* Initialized the weights from the original BERT model.

** Initialized the weights from the SciFive-PubMed-PMC model.

*** Initialized the weights from T5-Base model.

BioMedBERT was previously named “PubMedBERT”.

Among the generative models, T5-EHR v5 achieves the strongest overall performance, outperforming them on these biomedical benchmarks. T5-EHR v5 benefits from combining clinical and biomedical text with clinically aligned vocabulary. This combination appears to enhance the model’s ability to generalize across diverse biomedical relation tasks.

When compared with discriminative models, T5-EHR v5 performs competitively across all tasks. It matches or exceeds their performance on ChemProt and DDI, with the only exception where BioLinkBERT reports the highest score on GAD. However, as mentioned before, the GAD test set is considerably smaller than the others.

Overall, the analysis indicates that the T5-EHR models, despite being generative in nature, perform competitively with widely known discriminative models in these biomedical tasks. This suggests that the inclusion of diverse corpora like MIMIC, PubMed, and PMC helps the T5-EHR models maintain competitive performance levels in biomedical tasks, demonstrating reasonable generalization from clinical to broader biomedical domains.

## 6. Conclusions

Motivated by the question of how pretraining corpus and vocabulary choices affect the performance of high-performance language models, particularly in the clinical domain, we developed five variants of the T5-EHR model, each trained on different combinations of corpus and vocabulary. While a primary goal of our work was to develop high-performing models tailored for clinical tasks, we were also interested in evaluating their ability to generalize to broader biomedical applications.

Our experiments demonstrate that both corpus configuration and vocabulary selection are critical factors influencing the performance of T5-based models in clinical and biomedical domains. The results confirm that the best outcomes are typically achieved when the training data and vocabulary are well aligned with the target task. Notably, the choice of corpus and vocabulary appears to have a greater impact than simply increasing the size of the training data, as illustrated by the finding that adding PMC text does not consistently enhance performance on clinical tasks. Also, our results further emphasize a key distinction between biomedical and clinical NLP. Although large biomedical generative models gain from scale and extensive training on curated literature, strong performance on clinical tasks relies more heavily on alignment with real-world EHR language. This finding underscores the importance of targeted clinical pretraining and highlights the novelty of our work in systematically evaluating generative models trained from scratch on clinical corpora, rather than relying solely on biomedical pretraining.

In addition to our findings on the impact of corpus and vocabulary choices in clinical and biomedical domains, we have shown that the T5-EHR models outperform existing tools on most of the benchmark tasks we evaluated. We are making them publicly available on PhysioNet to support future research and provide a foundation for advancing generative modeling approaches in specialized healthcare domains.

Our findings demonstrate that, with an appropriate choice of pretraining data and vocabulary, generative models like T5-EHR can achieve top performance even on biomedical benchmarks, rivaling strong discriminative baselines. Beyond their competitive results, generative models offer distinct advantages for future applications such as clinical summarization and question answering, where output generation is essential.

Finally, T5-EHR generates predictions through a probabilistic decoder without explicitly modeling prediction uncertainty, which may lead to overconfident outputs in some cases. In addition, decoding strategies can bias generation toward frequent classes, and the decoder may reflect biases inherited from the underlying clinical and biomedical corpora. These considerations are particularly important for clinical applications and should be considered when interpreting model outputs and motivate future work on improving uncertainty awareness and reliability of generative models in clinical applications.

## Supporting information

S1 Appendix Additional methodological details, task descriptions, hyperparameter settings, and extended analyses [21–30]. (DOCX)
