# Supplementary material for: Pretraining effective T5 generative models for clinical and biomedical applications
Source: PLoS One. 2026 Apr 17;21(4):e0342610. doi: 10.1371/journal.pone.0342610 (PMC13089888; doi:10.1371/journal.pone.0342610)
Supplement: S1 Appendix — (DOCX) [file pone.0342610.s001.docx]

**Appendix**

**A: Corpora and Vocabulary Details**

To achieve our objectives, we utilized PubMed, PMC [4, 12], MIMIC III [21], and MIMIC IV [22] to pretrain our T5-EHR models from scratch. MIMIC-III and MIMIC-IV are comprehensive, publicly available databases containing de-identified health records of patients admitted to intensive care units (ICUs) at the Beth Israel Deaconess Medical Center. MIMIC-III covers data from 2001 to 2012, while MIMIC-IV extends this to include admissions up to 2019. MIMIC has been widely used to advance critical care research and develop machine learning models in healthcare due to their richness and accessibility. Both MIMIC-III and MIMIC-IV consist of over 2 billion tokens. The PubMed Abstracts dataset is a free resource comprising over 30 million citations and abstracts of biomedical literature, amounting to approximately 4.5 billion tokens. Additionally, the PMC Full-text articles dataset is an open-access collection of over 5 million full-text articles from biomedical and life science research, consisting of about 13.5 billion tokens. We preprocessed the text and split it into sentences following the methodology outlined by [11].

In addition, we built two different vocabularies, one constructed from MIMIC notes only and the other one from PubMed corpora. Both vocab dictionaries contain 30K tokens. We utilized the SentencePiece model [23] as a base vocabulary model.

**B: Downstream Task Details**

**B.1 Clinical Tasks:**

MedNLI [24]: is a specialized dataset curated and annotated by doctors to address the task of Natural Language Inference (NLI) within the medical domain. Each pair of sentences in the dataset is labeled as one of three possible outcomes: entailment, contradiction, or neutral.

i2b2-2010 RE [25]: the focus is on a relation extraction task designed to detect eight specific clinical events from electronic health records (EHRs). The goal is to accurately identify and extract relationships between various clinical entities, such as medical problems, treatments, and tests. Relation annotated are: Treatment improves medical problem (TrIP), Treatment worsens medical problem (TrWP), Treatment causes medical problem (TrCP), Treatment is administered for medical problem (TrAP), Treatment is not administered because of medical problem (TrNAP), Test reveals medical problem (TeRP), Test conducted to investigate medical problem (TeCP), Medical problem indicates medical problem (PIP), and no relation.

RadQA [26]: is a benchmark designed to evaluate the ability of models to perform question answering in the radiology domain. It leverages a carefully curated dataset composed of radiology reports and corresponding questions created by doctors.

CLIP [27]: The CLIP dataset is a curated collection of clinical action items annotated over the MIMIC-III by physicians and encompasses 718 discharge summaries, covering a total of 107,494 sentences. The dataset includes seven distinct labels representing different types of clinical actions that may be required: Appointment, Lab, Procedure, Medication, Imaging, Patient Instructions, and Other. Each sentence in the discharge summaries may be annotated with zero or more of these labels.

**B.2 Biomedical Tasks:**

ChemProt [28]: is a specialized benchmark focused on the extraction of chemical-protein interactions from biomedical literature. We follow the standard practice of evaluating the five most common classes.

DDI-2013 [29]: is a benchmark task designed to assess the ability of language models to identify and classify drug-drug interactions (DDIs) within biomedical literature. DDI-2013 distinguishes several types of interactions, including advice, effect, mechanism, and int (indicating other types of interactions).

GAD [30]: Gene-Disease Associations is a binary relation extraction benchmark focused on extracting and identifying associations between genes and diseases from biomedical literature.

**C: Fine-Tuning Hyperparameters**

We initially set the learning rate to 1e-3, as recommended by [2], but found it to be too high. Instead, a learning rate of 1e-4, as recommended by [3], yielded significantly better results across all tasks. We also experimented with different batch sizes [8,16,32] and observed that smaller batch sizes consistently led to improved performance. For most tasks, training for 10–20 epochs was sufficient for convergence. However, for RadQA, 60 epochs proved to be optimal.

Additionally, we performed a search over sequence lengths [128, 256, 512, 1024] and selected the best-performing length for each task. The optimal hyperparameters were determined by evaluating performance on the development set. Our hyperparameter search was conducted using T5-EHR v1, systematically exploring various combinations of learning rates, batch sizes, epochs, and sequence lengths. Once the best-performing hyperparameters were identified for each task, these settings were fixed and applied consistently across all models in our study to ensure a fair comparison and maintain evaluation consistency.

**D: Model Release and Fine-Tuning Resources**

All five T5-EHR model variants described in this study will be made available through the PhysioNet repository under credentialed access. Each model release includes the complete set of files required for reuse and reproducibility, including the model architecture configuration file (*config.json*), pretrained model weights in Flax format (*flax_model.msgpack*), the SentencePiece tokenizer model (*spiece.model*), and the generation configuration file used for inference (*generation_config.json*). Additional usage guidance and a README file describing each model variant and its associated vocabulary are also provided.

In accordance with PLOS ONE’s code-sharing policy, all author-generated fine-tuning scripts used in this manuscript will be made publicly available upon publication. These scripts will enable full reproduction of the reported experiments and facilitate adaptation of the released models to downstream clinical and biomedical NLP tasks.

**E: Out-of-Vocabulary (OOV) Rate Comparison for Clinical Tasks:**

| Task | Vocab | Total Tokens | OOV Tokens | OOV Rate | Coverage |
| --- | --- | --- | --- | --- | --- |
| MedNLI | PubMed | 365096 | 621 | 0.001701 | 0.998299 |
| MedNLI | MIMIC | 319353 | 8938 | 0.027988 | 0.972012 |
| i2b2-2010 RE | PubMed | 1453813 | 89904 | 0.061840 | 0.938160 |
| i2b2-2010 RE | MIMIC | 1302286 | 52824 | 0.040563 | 0.959437 |
| RadQA | PubMed | 720583 | 5476 | 0.007599 | 0.992401 |
| RadQA | MIMIC | 619934 | 10301 | 0.016616 | 0.983384 |
| CLIP | PubMed | 1662649 | 5397 | 0.003246 | 0.996754 |
| CLIP | MIMIC | 1443128 | 21636 | 0.014992 | 0.985008 |


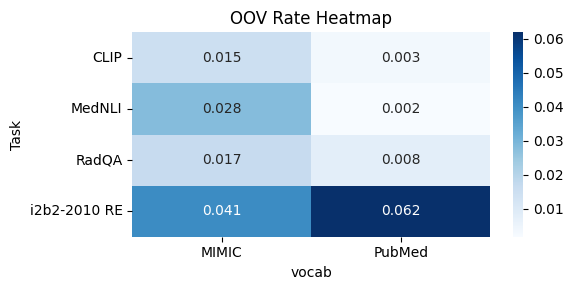


**F: Per-Class Performance and Imbalance Analysis for Clinical Tasks:**


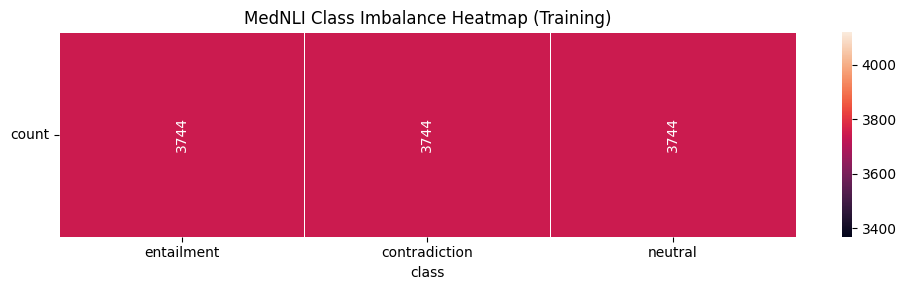


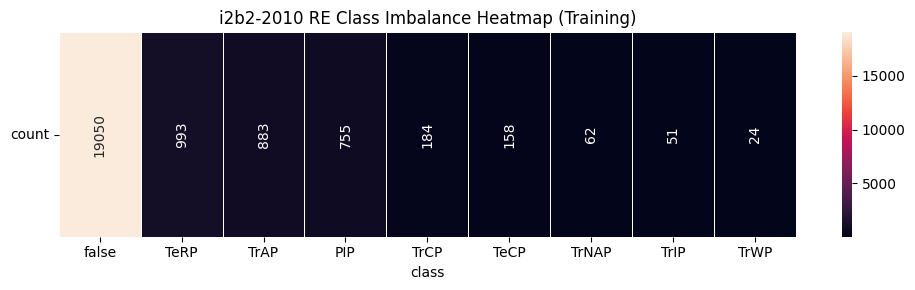


**
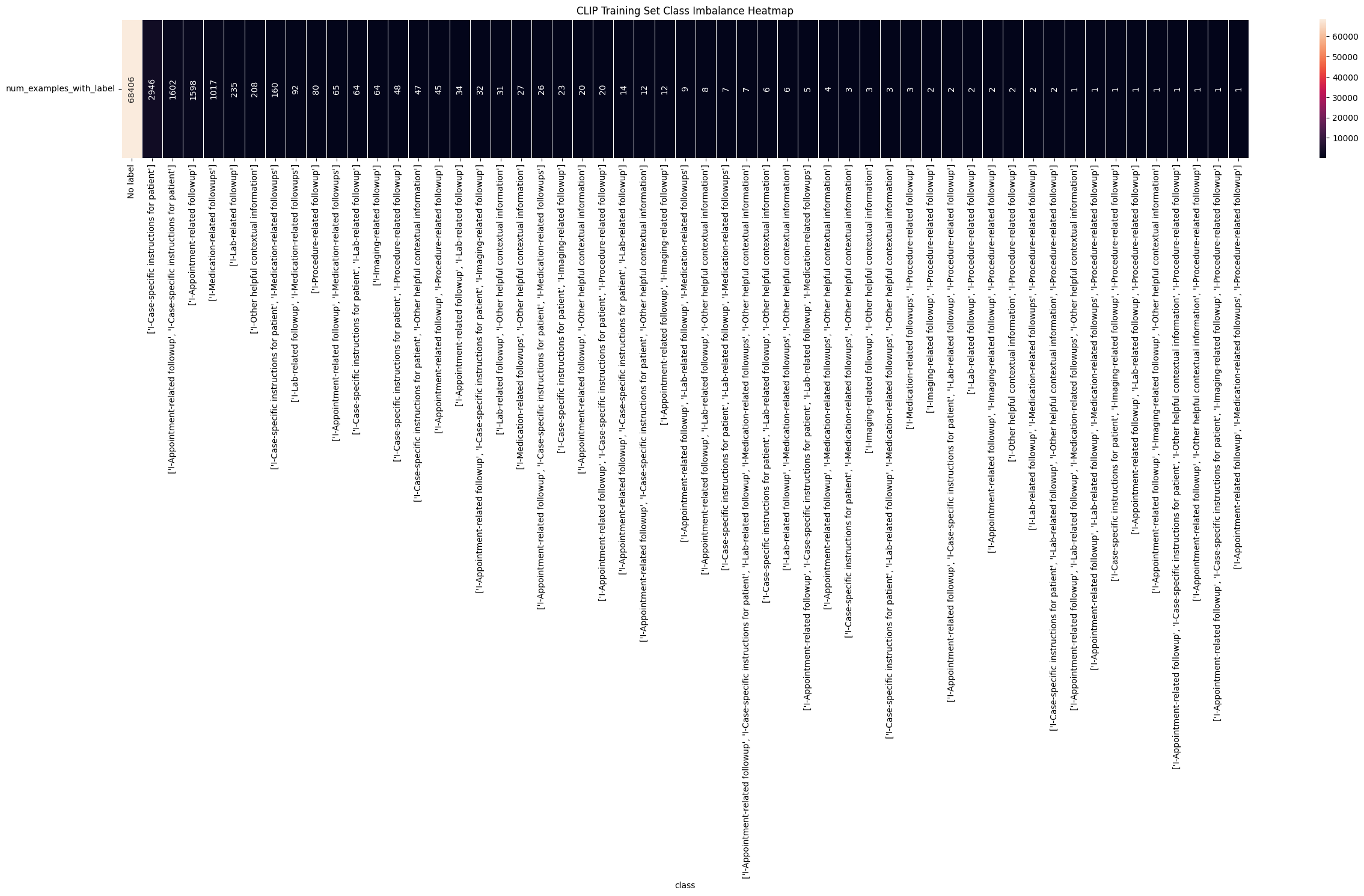
**
